# A randomised, phase II, unblinded trial of an Exercise and Nutrition-based Rehabilitation programme (ENeRgy) versus standard care in patients with cancer: feasibility trial protocol

**DOI:** 10.1186/s40814-018-0381-6

**Published:** 2018-12-27

**Authors:** Charlie C. Hall, Lucy Norris, Liz Dixon, Jane Cook, Matthew Maddocks, Catriona Graham, Sharon Tuck, Erna Haraldsdottir, Duncan Brown, Anna Lloyd, Anne Finucane, Peter Hall, Katharina Diernberger, Richard J. E. Skipworth, Marie Fallon, Barry J. Laird

**Affiliations:** 1 St Columba’s Hospice, 15 Boswall Road, Edinburgh, UK; 2 Institute of Genetics and Molecular Medicine, University of Edinburgh, Edinburgh, UK; 3 Southampton Clinical Trials Unit, University of Southampton, Southampton, UK; 4 Cicely Saunders Institute of Palliative Care, Policy and Rehabilitation, Kings College London, London, UK; 5 Epidemiology and Statistics Core, Edinburgh Clinical Research Facility, University of Edinburgh, Edinburgh, UK; 6 Marie Curie Hospice, 45 Frogston Road West, Edinburgh, UK; 7 School of Medicine and Veterinary Medicine, Edinburgh Cancer Research UK, University of Edinburgh, Edinburgh, UK; 8 Department of Clinical Surgery, Royal Infirmary, Edinburgh, UK

## Background

Patients are living longer with incurable cancer [1] such that in many cases, cancer is likened to a chronic disease [2–4]. This development has wide-ranging implications for both patients and wider society, with increased longevity comes increased morbidity and associated socio-economic burden [5, 6]. Primary cost drivers for patients with advanced cancer are hospitalisation, GP and domiciliary visits [7]. Rehabilitation has been advocated as one such way of optimising the function and quality of life in this group of patients [8]; however, the optimal components of a rehabilitation model for patients with incurable cancer remain to be elucidated.

In the past, there was a therapeutic nihilism that functional decline, cachexia and psychological distress were inevitable consequences of cancer [9, 10]. This is no longer the case and differs markedly from the modern palliative care approach, where advancements in symptom management, embraced in holistic care, have made dramatic improvements in the care of patients over the past 30 years. However, this progress has been slow to incorporate rehabilitation; indeed optimising physical function and nutritional status has largely been ignored [11]. Patients, their families and clinicians realise that optimising quality of life is a fundamental component of good cancer care and that maintaining physical function and nutrition is as important as good symptom control [12]. Although clear guidance exists on symptom control, programmes which optimise physical and nutritional function have been the exception rather than the norm.

The concept of rehabilitation is widely established for the management of chronic diseases such as chronic respiratory disease [13]; yet in palliative care, the concept of rehabilitation remains largely elusive. Rehabilitation in the context of patients with incurable cancer aims to improve function where there is a capacity to do so, to maintain function where the effects of the illness or its treatment threaten to cause decline and to ease the transition toward functional decline where deterioration is inevitable [14].

In 2015, Hospice UK published a report advocating that “rehabilitative palliative care is an essential component of palliative care” [8]. This comprehensive report argued that rehabilitation should focus on function, be person-centred and enable patients to live fully by maintaining or adapting their functional independence while supporting self-management. Guidance was also offered on how rehabilitation should be implemented in the UK, including adopting and embedding a culture of rehabilitative palliative care. However, there is limited robust evidence on which to base this approach. A recent systematic review examining rehabilitation in advanced cancer identified only a small number of studies in this area [4]. Evidence suggests that rehabilitation may be feasible for patients with advanced cancer, but key components are not clear and no firm recommendations could be given. A further review has highlighted the lack of studies examining combined exercise and nutrition interventions for patients with advanced cancer [11].

While there is evidence of the benefits of rehabilitation in non-malignant conditions, such as chronic respiratory disease [13], extrapolating these models to incurable cancer care needs evaluation. The majority of work to date in patients with incurable cancer has focused on exercise as a single intervention [15]. Although exercise is important, it has been argued that any rehabilitation programme in incurable cancer should also focus on nutritional aspects [11]. This would seem logical as approximately 20% of cancer deaths are directly attributable to cancer cachexia, and cachexia is highly prevalent in patients with advanced cancer [9, 16]. Cachexia is the multifactorial syndrome, defined by ongoing loss of skeletal muscle mass (with or without loss of fat mass) that cannot be fully reversed by conventional nutritional support, causing progressive functional impairment [17]. Optimising nutrition is fundamental to facilitate post-prandial anabolism, which is key to maintaining muscle and thus physical function [18]. There is a persuasive argument that exercise and nutrition should be considered as cornerstones of rehabilitation programmes in patients with incurable cancer [19]. However, this remains to be demonstrated in clinical practice.

Previous studies have demonstrated the detrimental effect of deteriorating physical function on survival [20]. It therefore follows that optimising physical function may have survival benefits. At the very least, it may enable patients to remain independent for longer periods. Previous work by our group has examined an exercise and nutrition-based intervention in oncology outpatients with lung and pancreatic cancer undergoing chemotherapy and demonstrated that such an intervention was feasible and had beneficial effects on physical function and weight [21]. A recent randomised control trial has shown good adherence to an exercise and nutritional intervention in palliative lung and gastrointestinal cancer patients, with beneficial effects on symptoms of nausea and vomiting and protein intake [22].

These findings are encouraging; however, the potential benefits of an exercise and nutrition-based rehabilitation programme in a general population of patients with incurable cancer remain unclear. The ENeRgy trial aims to determine whether an exercise and nutritional rehabilitation programme is feasible in a hospice outpatient setting for patients with incurable cancer. It aims to also examine changes in physical function, nutritional status and quality of life in these patients. Effects on partner-carer quality of life as well as healthcare resource utilisation will also be examined. A companion qualitative study, ‘ENeRgy-Q’, will be undertaken to explore acceptability, compliance and the psychosocial impact of this rehabilitation programme for patients with incurable cancer in the hospice setting.

## Methods

### Design

This is a randomised, unblinded feasibility trial of an Exercise and Nutrition-based Rehabilitation programme (ENeRgy) versus standard care in patients with incurable cancer. Full ethical approval has been given (17/WS/0226), and the trial will be conducted according to principles of Good Clinical Practice and the Declaration of Helsinki.

### Population

**Fig. 1:**
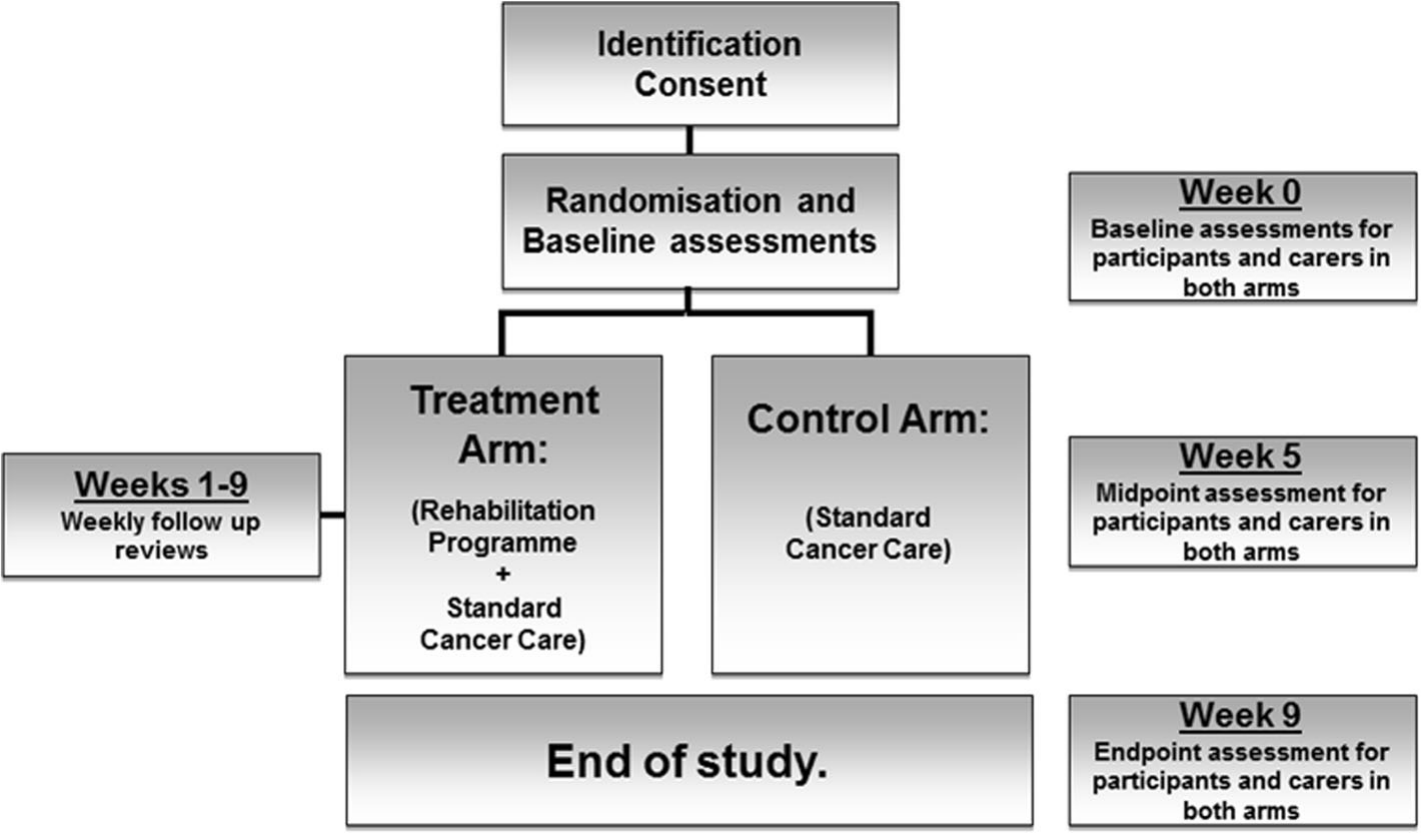
Trial schematic

Eligible patients will meet the following key criteria: > 18 years of age, Karnofsky Performance Status (KPS) > 60, diagnosis of incurable cancer (defined as metastatic or locally advanced cancer not amenable to curative treatment), not undergoing anti-cancer therapy (hormonal treatment or bisphosphonates permitted) with a prognosis greater than 3 months. Eligible patients are community-dwelling and have the capacity to consent and complete trial-based assessments. Participants will be identified and referred to the trial from one of two hospice community palliative care teams or from the regional oncology service. Patients undergoing anti-cancer therapy (excluding hormone or bisphosphonate treatments), using enteral nutrition, unable to swallow or co-enrolled in drug trials are excluded. Figure 1 details the trial schematic. The consent process will be opt in, and written informed consent will be obtained by the trial research nurse or doctor. After baseline assessments (which occur over 7 days; week 0), patients will be randomised (1:1 stratified by baseline KPS 60–80%, 90–100%) to receive either an 8-week exercise and nutrition-based rehabilitation programme (treatment arm) or standard care (control arm). Patients randomised to the control arm will be offered the study intervention after trial completion.

The trial is being conducted in a single centre (a hospice) serving a geographically defined region in the UK with a population of approximately one million. Trial-related assessments will take place at an outpatient clinic at this hospice. Management of the trial will be overseen by a Trial Management Group (TMG). Patient and public involvement (PPI) for the trial has been provided by Marie Curie’s Expert Voices group, as well as an ex-carer of a patient with cancer. PPI input has been highly valued, ranging from design of trial documents to regular presence at TMG meetings.

### Interventions

#### Treatment arm

The treatment arm is an exercise and nutrition-based rehabilitation programme. Patients allocated to this arm will have an interview with the trial physiotherapist and dietitian at week 1. They will then be given an individualised exercise and nutrition-based rehabilitation programme following this assessment. Key components of this include the following:

##### Exercise

A home-based exercise programme is supported by a booklet. This will consist of aerobic and resistance exercise in divided sessions of the patient’s choosing. The aerobic component comprises a total of 60 min of physical activity over the course of each week at moderate intensity, i.e. feeling warm and getting slightly out of breath (able to talk), equivalent to an intensity of 3–4 rating of perceived exertion on a modified Borg scale [23]. Walking will be recommended as the main type of physical activity although cycling or more vocational forms of activity, e.g. heavy housework and gardening, can be used as long as they provoke the desired level of exertion. The resistance component involves major muscle groups in the upper and lower body (e.g. half squats, standing press-ups, shoulder press) and will be recommended three times per week. Patient diaries will record the amount of resistance and aerobic exercise taken daily and any difficulties with particular exercises.

##### Nutrition

The main goal of the nutritional intervention is to promote energy balance and to ensure optimal nutritional intake. The nutritional component consists of individual dietary counselling to enhance overall dietary intake [19, 21] and oral nutritional supplements (ONS).

Individual dietary counselling will continue weekly throughout the trial by the trial dietitian. Dietary advice will be tailored and take into account any specific requirements, e.g. ethnic background. Patients will be instructed to take two ONS per day. One ONS portion (220 mL) contains 1 g of eicosapentaenoic acid (EPA), and the caloric distribution is relevant for cancer patients experiencing unintended weight loss. Patients not able to tolerate the ONS due to personal preference will be offered an alternative ONS plus capsules containing 2 g EPA. Patient information leaflets will detail varied ways to take the ONS to improve compliance, and diaries will record the numbers of ONS taken daily.

At weekly review appointments, patient diaries will be reviewed by the research nurse for healthcare-related resource use; adverse events will also be screened for and logged. The trial dietitian will review the patients’ dietary intake and compliance with the ONS, and the trial physiotherapist will review exercise progress, offer goal setting and prompt any changes needed to maintain compliance.

#### Control arm

Patients randomised to the control arm will continue to receive standard care from their GP and community palliative care team on an as required basis according to individual patient need. This care may also include referral to other members of the community-allied healthcare professional MDT team if required (for example counsellors, occupational therapist or social workers). The control group will be phoned at weekly intervals by the research nurse to ascertain levels of healthcare-related utility and adverse events. In the control group, patients will also have diaries to record any (non-trial) nutritional supplements they are taking as well as the amount and type of exercise undertaken each week. This will help gauge any degree of contamination in the control group.

Patients in the control arm will be offered the opportunity to undertake the rehabilitation programme at the end of their involvement in the trial if they wish to do so.

### Outcomes

The primary endpoint is to evaluate the feasibility of delivering the exercise and nutritional rehabilitation programme in a hospice outpatient context. This will be assessed by measuring compliance with the rehabilitation programme (numbers of exercises and nutritional supplements versus those advised). Compliance with trial procedures will also be measured, including completion of diaries and questionnaires, percentage withdrawal, completion of physical tests and completeness of physical activity monitor data.

Secondary endpoints will examine the feasibility of recruitment and retention, evidence of contamination in the control group and change in physical function and nutritional status. Quality of life measures for patients (± partner-carers) and impact on patient healthcare-related resource use in terms of cost between sectors of the NHS, social services, third sector, participant expenses and carer costs will also be examined. All endpoints will be assessed at baseline (pre-randomisation—week 0) and at trial endpoint (week 9). Table 1 details a summary of trial-related assessments and time points.

**Table 1:** Trial-related assessments and time points (both arms)

	Baseline measures (week 0)	Midpoint (week 5)	Endpoint (week 9)
Demographics	• Gender, primary tumour site and tumour status; metastatic sites; current hormone/bisphosphonate or steroid treatment	• N/A	• N/A
Physical measures	• Height • Weight	• Weight	• Weight
Quality of life (QOL) measures	• Patient QOL (EORTC QLQ-C15-PAL questionnaire) [24] • Partner-Carer QOL^a^ (Caregiver Quality of Life Index-Cancer Questionnaire (CQOLC)) [25] • EQ-5D-5L & EQ-VAS [26] questionnaires	• Patient QOL (EORTC QLQ-C15-PAL) • Partner-Carer QOL^a^ (CQOLC) • EQ-5D-5L & EQ-VAS questionnaires	• Patient QOL (EORTC QLQ-C15-PAL) • Partner-Carer QOL^a^ (CQOLC) • EQ-5D-5L & EQ-VAS questionnaires
Functional measures	• Karnofsky Performance Status (KPS) [27] • Life Space Assessment questionnaire (LSA) [28] • Two-minute walk test [29] • Timed up and go test [30]	• KPS • LSA • Two-minute walk test • Timed up and go test	• KPS • LSA • Two-minute walk test • Timed up and go test
Socio-economic measures	• Socio-economic background (employment status, benefits received, carer responsibilities, current use of social services) • Healthcare utilisation and expenses questionnaire	• Healthcare utilisation and expenses questionnaire	• Healthcare utilisation and expenses questionnaire
Physical activity meter (PAM)	• PAM worn continuously for 7 days^b^ (data retrieved at week 1) • Mean daily step count • Hours asleep/ restless/ awake per night	(PAM worn only at baseline and end point)	• PAM worn continuously for 7 days^b^ (data retrieved at week 10) • Mean daily step count • Hours asleep/restless/awake per night
Nutritional measures	• Abridged Patient-Generated Subjective Global Assessment (aPG-SGA) [31] • Ten-point verbal analogue scale (AveS) [32]	• aPG-SGA • AveS	• aPG-SGA • AveS

^a^‘Partner-carer’ is a partner with whom the patient is married, cohabiting or non-cohabiting, and the patient also describes as their carer

^b^PAM data for weekend days may be excluded to reduce potential variation

### Statistical considerations

The primary endpoint of this study is to assess the feasibility of the treatment (an exercise and nutrition-based rehabilitation programme). As such, a formal sample size calculation has not been performed. We plan to recruit over a 13-month period and expect to be able to obtain at least 40 participants over that timeframe. Intention-to-treat analysis will be performed.

The primary outcome measures will be presented descriptively using appropriate summary statistics with corresponding 95% confidence intervals. Demographic statistics and exploratory outcome measures shall also be presented using appropriate summary split by treatment group. Continuous outcome measures, for example, change in daily step count and change in weight, will be compared between treatment arms using two sample *t* tests or non-parametric equivalent as appropriate. Rates of compliance will be reported along with completion rates for all other outcome measures. This feasibility trial is not powered to explore efficacy, but these estimates of variability will be used to inform the sample size and inform our choice of primary endpoint for the definitive trial. There are no plans to perform an interim analysis while recruitment is ongoing or before follow-up is completed. Estimation of economic parameters will rely on questionnaires designed to measure health-related utility, healthcare-related resource use and costs, administered at baseline and follow-up assessment time points. Unit costs will be assigned using standard national costing sources where available or through consultation with relevant service business managers. Costs will be summarised from the perspectives of (a) the NHS, (b) the charitable and 3rd sector, (c) the patient and their carers and (d) wider society. A proof-of-concept health economic model will be constructed taking the form of a probabilistic decision model that simulates the passage of patients through the clinical pathway defined by discrete health states, allowing estimation of costs, quality of life and survival. The model will be parameterised using data from the feasibility trial where possible, supplemented by data from the published literature. Cost-effectiveness will be presented as the incremental cost-effectiveness ratio (ICER), expressed as cost per QALY gained.

A computer-generated randomisation schedule will be produced using a random block size to allocate patients at random in a 1:1 ratio to either the treatment arm (personalised exercise and nutrition-based rehabilitation programme) or control arm (standard care) via sealed envelopes. The randomisation will be stratified by performance status due to its influence on prognosis to ensure that patients with differing prognoses are equally distributed between arms (KPS of 60–80% versus KPS 90–100%). Randomisation will occur at baseline (week 0) but will be blinded to patients until week 1 when it will be revealed by the research nurse so as not to influence baseline activity levels in either group during baseline assessments.

Paper case report forms (pCRF) will be used, and data will be entered directly into an electronic data base. A 10% check will be undertaken on all inputted data to ensure validity. Patients will be identified by a unique trial identification number, and patient identifiable data will be kept locked securely within the hospice. Standard operating procedures (SOPs) issued by the trial sponsor (ACCORD/NHS Lothian) will be adhered to for example reporting deviations from the protocol or serious adverse events (SAEs).

## Discussion

One of the fundamental arguments supporting rehabilitation is the changing face of cancer. Although initially regarded as a terminal disease, cancer is morphing into a chronic condition which in combination with its increasing incidence will mean that more patients are ‘living with’ rather than ‘dying from’ their cancer. Combined with an ageing population, this means that the population who would fit under the umbrella of palliative care is likely to rise considerably over the coming decades. It is important that in view of this potential increase in patient numbers, the overall condition of patients is optimised through maximisation of physical function and nutritional status.

The ENeRgy trial is a key step in defining, developing and assessing the feasibility of an exercise and nutrition-based rehabilitation programme in this patient cohort. We will use the trial to test the mechanism of healthcare resource use data capture with a view to identifying key possible drivers of cost differences. The results of this trial and subsequent studies have the potential to significantly impact and influence the approach to rehabilitation for patients with incurable cancer in the future.

### Trial status

The description of the trial is in keeping with the approved version of the trial protocol (version 3, date 15 April 2018). The trial has been open to recruitment from 30 January 2018, and recruitment is expected to last 13 months, ending on 28 February 2019.

## Abbreviations

aPG-SGA: Abridged Patient Generated Subjective Global Assessment
AveS: Ten-point verbal analogue scale for dietary intake
CQOLC: Caregiver Quality of Life Index- Cancer Questionnaire
ENeRgy: Exercise and Nutrition-based Rehabilitation programme (ENeRgy)
EORTC QLQ-C15-PAL: A questionnaire developed to assess the quality of life of palliative cancer care patients
EPA: Eicosapentaenoic acid
EQ-5D-5L & EQ-VAS: Euroqol questionnaires regarding quality of life. The 5D is five level descriptive questionnaire and the VAS is a visual analogue scale
GP: General practitioner
KPS: Karnofsky Performance Status
LSA: Life Space Assessment questionnaire
ONS: Oral nutritional supplement
PAM: Physical Activity Monitor
pCRF: Paper case report form
QOL: Quality of Life
SAEs: Serious adverse events
SOPs: Standard operating procedures
